# Inhibitors of RNA and protein synthesis cause Glut4 translocation and increase glucose uptake in adipocytes

**DOI:** 10.1038/s41598-022-19534-5

**Published:** 2022-09-19

**Authors:** A. B. Meriin, N. Zaarur, J. S. Bogan, K. V. Kandror

**Affiliations:** 1 Department of Biochemistry, Boston University School of Medicine, Boston, MA 02118 USA; 2 Department of Internal Medicine and Cell Biology, Yale University School of Medicine, New Haven, CO 06520 USA

**Keywords:** Biochemistry, Cell biology

## Abstract

Insulin stimulates glucose uptake in adipocytes by triggering translocation of glucose transporter 4-containg vesicles to the plasma membrane. Under basal conditions, these vesicles (IRVs for insulin-responsive vesicles) are retained inside the cell via a “static” or “dynamic” mechanism. We have found that inhibitors of RNA and protein synthesis, actinomycin D and emetine, stimulate Glut4 translocation and glucose uptake in adipocytes without engaging conventional signaling proteins, such as Akt, TBC1D4, or TUG. Actinomycin D does not significantly affect endocytosis of Glut4 or recycling of transferrin, suggesting that it specifically increases exocytosis of the IRVs. Thus, the intracellular retention of the IRVs in adipocytes requires continuous RNA and protein biosynthesis de novo. These results point out to the existence of a short-lived inhibitor of IRV translocation thus supporting the “static” model.

## Introduction

Elevated circulating glucose represents a hallmark of type 2 diabetes mellitus that leads to numerous diabetes complications, such as neuropathy, retinopathy, amputations, etc.^1^. In mammals, clearing of blood glucose is achieved by insulin-dependent translocation of the glucose transporter isoform 4, Glut4, to the plasma membrane of fat and skeletal muscle cells. Multiple lines of independent evidence including various transgenic models^2–4^ and in vivo NMR studies^5^ have proved that Glut4-mediated glucose uptake represents a rate-controlling step of not just blood glucose control, but of overall insulin-stimulated glucose disposal.

In basal adipocytes, about 50% of all intracellular Glut4 is localized in insulin-responsive vesicles, or IRVs. The rest of the transporter is distributed between various intracellular compartments that are not usually considered insulin-sensitive, such as endosomes, trans-Golgi network, and ubiquitous transport vesicles that shuttle between different intracellular compartments^6,7^. Less than 5% of Glut4 is localized in the plasma membrane, and even smaller amounts in the biosynthetic pathway as Glut4 is normally a very stable protein and does not need constant replenishment by de novo biosynthesis.

How exactly insulin triggers translocation of the IRVs is not known. The best studied signaling pathway involves PI 3 kinase, Akt, TBC1D4, and Rab10^8,9^. However, the mechanism of TBC1D4/Rab10 action is still unknown. These proteins may induce formation of the IRVs, their translocation to or fusion with the plasma membrane. Importantly, TBC1D4/Rab10-mediated signaling accounts for approximately 50% of the total effect of insulin on glucose uptake in adipocytes^8^ leaving room for alternative or additional effectors.

Other PI 3 kinase-dependent and independent mechanisms of Glut4-mediated glucose uptake have been described (reviewed in^10^). For example, insulin activates a PI 3 kinase-independent TC10α-PIST pathway that causes the proteolytic cleavage of the IRV anchor TUG by the protease USP25m^11–13^. It is not fully understood whether various insulin signaling pathways converge on the same target and/or regulate different vesicle trafficking steps. Although it has been shown that TBC1D4 is present on TUG-bound vesicles^13^, the integration of multiple non-overlapping pathways of insulin signaling into one general model remains, at present, uncertain.

It has also been shown that insulin administration to adipocytes causes dramatic changes in proteostasis. In particular, insulin increases general protein synthesis and enhances protein stability^14^. Whether or not the effect of insulin on Glut4 translocation and glucose uptake requires alterations in the adipose proteome is unknown. We decided to explore this question and obtained an unexpected answer. We have found that inhibitors of RNA and protein synthesis stimulate translocation of Glut4 and glucose uptake in cultured 3T3-L1 adipocytes without engaging Akt, TBC1D4, or TUG. Our results indicate the existence of a critically important and relatively short-lived protein(s) that may function as a long sought-after intracellular anchor for the IRVs. We hypothesize that various seemingly independent effectors of Glut4 translocation may functionally inactivate this putative anchor via different mechanisms, such as post-translational modifications (i.e. Akt-dependent phosphorylation) and/or proteolytic degradation. This hypothesis may help to bring together various pathways of insulin signaling to Glut4-vesicles into one general model.

## Materials and methods

### Reagents and antibodies

Human insulin and emetine and α-amanitin were purchased from Sigma-Aldrich (St. Louis, MO); cytochalasin B, BODIPY 493/503, actinomycin D—from Cayman Chemical (Ann Arbor, MI) and fetal bovine serum (FBS)—from Atlanta Biologicals (Lawrenceville, GA). Calf bovine serum (CBS), ProLong® Gold antifade reagent with DAPI, Halt™ Protease and Phosphatase Inhibitor Cocktail, puromycin, DMEM and DPBS—from Thermo Fisher Scientific (Waltham, MA). Deoxy-D-glucose, 2-[1,2-3H(N)] was purchased from American Radiolabeled Chemicals (St. Louis, MO). Mouse monoclonal antibodies against actin and myc tag, rabbit monoclonal antibody against phospho-TBC1D4 (Thr642), rabbit polyclonal antibodies against the myc tag, phospho-Akt (Ser473 or Thr308), TBC1D4, HRP-conjugated anti-mouse and anti-rabbit IgG were purchased from Cell Signaling Technology (Danvers, MA). Mouse monoclonal anti-syntaxin 6 antibody was purchased from BD Biosciences (Franklin Lakes, NJ). Mouse monoclonal antibody 1F8 against Glut4 was a kind gift of Dr. Paul Pilch. Rabbit polyclonal antibody against TUG were described previously^11,12^. Mouse monoclonal antibody against puromycin was purchased from EMD Millipore (Burlington, MA). Alexa Fluor 594-conjugated goat anti-mouse antibody, Alexa Fluor 488-conjugated goat anti-rabbit antibody and Alexa Fluor 647-conjugated mouse transferrin were purchased from Jackson ImmunoResearch (West Grove, PA).

### Cell Lines

3T3-L1 cells were obtained from Zen-Bio (Durham, NC). 3T3-L1 cells stably transfected with pBabe-myc_7_-Glut4 were described previously^15^. 3T3-L1 fibroblasts were grown in DMEM containing 10% CBS. Three days after confluence, cells were transferred to the differentiation medium (DMEM with 10% FBS, 0.174 μM insulin, 1 μM dexamethasone, and 0.5 mM 3-isobutyl-1-methylxanthine). Three days later, differentiation medium was replaced with DMEM containing 10% FBS for 4 more days until over 90% of differentiation was achieved.

### Immunofluorescence

Differentiated 3T3-L1 adipocytes were plated on coverslips coated with poly-lysine (Neuvitro Corporation, Vancouver, WA) on the day preceding the experiment. Cells were fixed with 4% methanol-free formaldehyde in DPBS for 12 min, blocked with 5% goat serum at 4 °C overnight, and stained with primary antibodies for 2 h at room temperature followed by 1 h incubation with secondary antibody. Then, the cells were stained with BODIPY and DAPI, mounted on slides with the ProLong Gold antifade reagent and examined with the Axio Observer Z1 fluorescence microscope equipped with the Hamamatsu digital camera C10600/ORCA-R2 and Axiovision 4.8.1 program (Carl Zeiss Inc., Thornwood, NY).

Each microscopic image shows a representative result of at least two independent experiments. In each experiment, cells were counted on at least 5 randomly chosen fields. For quantifications, the intensity of all images was reduced to the level, at which signal from the non-specific control (i.e. cells stained with irrelevant primary antibody or without primary antibody) became undetectable. Scoring was blindly verified by an independent operator.

### Endocytosis of myc_7_-Glut4

Differentiated adipocytes stably expressing myc_7_-Glut4^15^ were plated on glass-bottom plates (Cellvis, Mountain View, CA) coated with poly-lysine. Next day, cells were treated with ActD and insulin. At the end of each treatment, cells were incubated with anti-myc monoclonal antibody (0.8 µg/ml) in DMEM for 1 h on ice, washed twice with cold DMEM, and either immediately fixed with 4% formaldehyde, or transferred to pre-warmed (37 °C) DMEM for 5 min before fixation. Cells were fixed with cold 4% formaldehyde in DPBS for 5 min on ice, and then fixation continued at room temperature for 15 more minutes. Cells were permeabilized with 0.2% Triton X-100 for 5 min, blocked with 5% goat serum overnight and stained with Alexa Fluor 594-conjugated anti-mouse secondary antibody.

### Recycling of transferrin

To measure endocytosis of transferrin, differentiated 3T3-L1 adipocytes were incubated with Alexa Fluor® 647 mouse transferrin (25 µg/ml) in DMEM for 7.5 or 15 min at 37 °C, washed twice with DPBS at 37 °C, fixed, and analyzed by immunofluorescence at the same exposure. To measure exocytosis of transferrin, differentiated 3T3-L1 adipocytes treated or not treated with ActD were incubated for 4 h with Alexa Fluor® 647 mouse transferrin (25 µg/ml) in DMEM at 37 °C, washed twice with DMEM with 10% FBS, incubated in the same medium for 10 and 20 min at 37 °C, fixed and stained with DAPI. Percentage of transferrin-containing cells was determined by immunofluorescence at the same exposure.

### [^3^H] 2-Deoxyglucose uptake

The assay was performed in 6-well plates as described in^16^. Briefly, cells were washed three times with DMEM, starved in the same medium for 2 h, washed twice with warm (37 °C) Krebs–Ringer-HEPES (KRH) buffer without glucose (121 mM NaCl, 4.9 mM KCl, 1.2 mM MgSO_4_, 0.33 mM CaCl_2_, 12 mM HEPES, pH 7.4) and incubated with or without 100 nM insulin at 37 °C for 13 min. Radioactive 2-deoxyglucose was added to adipocytes for 5 min. The assay was terminated by aspirating the radioactive media, and the wells were washed four times with 2 ml of ice-cold KRH containing 25 mM D-glucose. Then, 400 μL of RIPA buffer (150 mM NaCl, 0.5% Sodium Deoxycholate, 0.1% SDS, 1% NP-40P, 50 mM Tris–HCl, pH 7.4) was added to each well. The lysates were transferred to Eppendorf tubes, subjected to ultra sonication, and 300 μl aliquots were removed for determination of radioactivity by liquid scintillation counting. Measurements were made in triplicates. Nonspecific diffusion assessed in the presence of 5 μM cytochalasin B, which was < 5% of the total uptake, was subtracted. The protein concentration in the lysates was determined using the BCA protein assay kit (Thermo Fisher Scientific, Waltham, MA) and was used to normalize counts. All 2-deoxyglucose uptake experiments have been repeated at least three times.

### Gel electrophoresis and Western blotting

Proteins were separated in SDS–polyacrylamide gels and transferred to nitrocellulose membranes (Bio-Rad Laboratories, Hercules, CA). The membranes were blocked with 3% BSA in TBST buffer with 0.1% Tween-20 for 30 min and cut prior to hybridization with various antibodies using pre-stained markers as guides. Membrane strips were probed overnight with specific primary antibodies at 4 °C followed by 1 h incubation at room temperature with horseradish peroxidase-conjugated secondary antibodies. Protein bands were detected with the enhanced chemiluminescence substrate kit (Pierce ECL Western Blotting Substrate, Thermo Fisher Scientific, Waltham, MA) using X-ray films and/or a Bio-Rad ChemiDoc™ XRS + System (Hercules, CA). Scanned images were cropped for the sake of space. Unprocessed images are shown in Supplemental Data. All Western blotting images are representative of at least two independent experiments.

## Results

**Figure 1:**
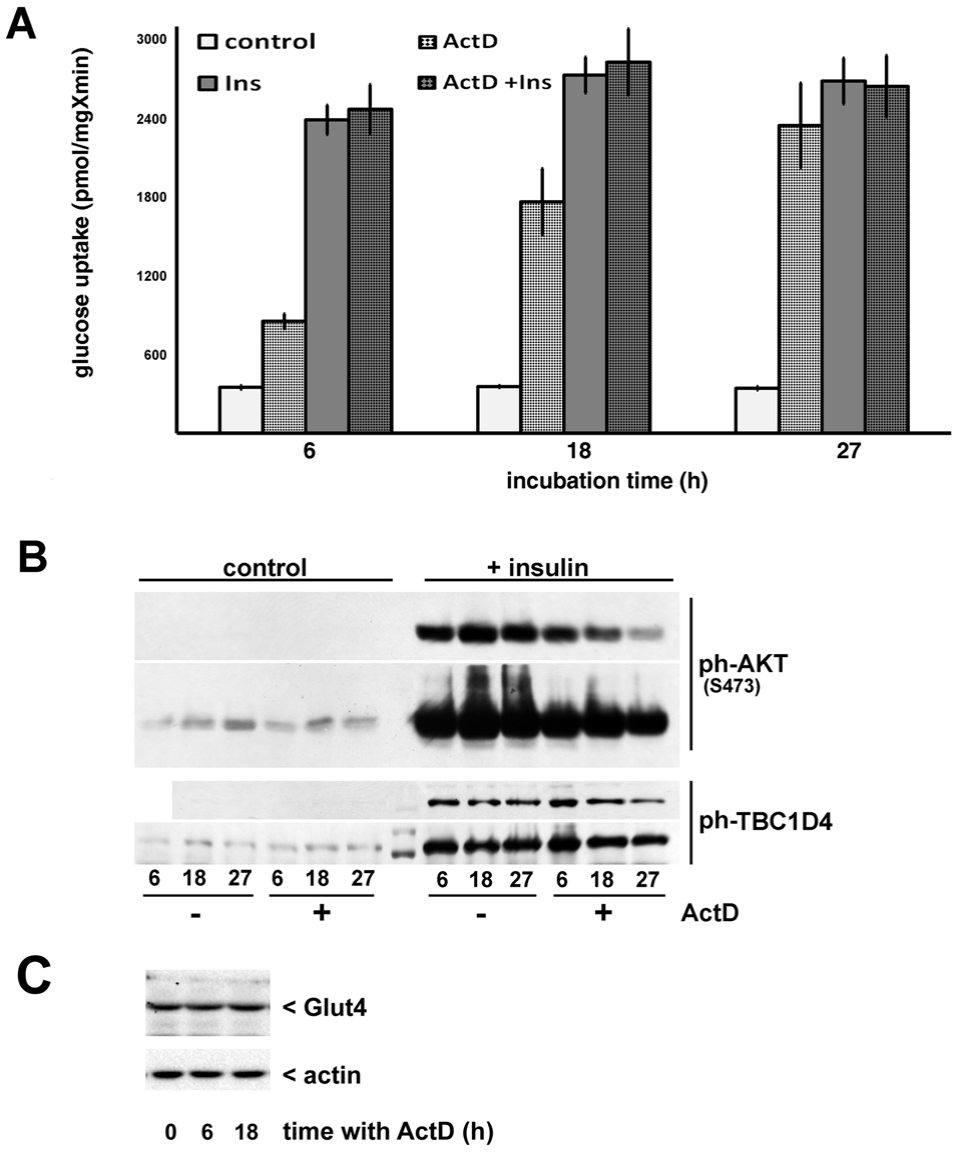
Actinomycin D increases glucose uptake in adipocytes without engaging the insulin signaling pathway. ActD (5 µM) was added to differentiated 3T3-L1 adipocytes for the indicated amount of time and cells were assayed for glucose uptake in the absence and in the presence of insulin (Panel** A**) or analyzed by Western blotting (Panels** B** and** C**). The error bars represent standard deviations. Panel B shows two different exposures.

In order to assess whether glucose uptake in 3T3-L1 adipocytes depends on continuous gene expression, we have incubated cells with the transcription inhibitor actinomycin D (ActD). We observed that ActD strongly increases glucose uptake in basal 3T3-L1 adipocytes in a time-dependent fashion. After 27 h incubation, the effect of ActD is virtually indistinguishable from that of insulin (Fig. 1A). Interestingly, the effects of insulin and ActD on glucose uptake in adipocytes are not additive; we suggest therefore that the insulin signaling pathway may involve an ActD-sensitive component(s). Furthermore, ActD does not trigger phosphorylation of Akt and TBC1D4 or markedly interfere with the effect of insulin on phosphorylation of these proteins (Fig. 1B; for original files see Fig. S1) indicating that ActD treatment may affect downstream element(s) of the signaling pathway(s). Even so, we have noticed that after 27 h, ActD attenuates insulin-stimulated phosphorylation of Akt and TBC1D4. Therefore, for further experiments, we used an 18 h long incubation with ActD in order to avoid potential artifacts. Note, that incubation with ActD for up to 18 h does not change intracellular Glut4 levels (Fig. 1C). We have confirmed that 18 h and 27 h long incubation of 3T3-L1 adipocytes with another inhibitor of transcription, *α*-amanitin, also increases basal glucose uptake (Fig. S2A) without activating the insulin signaling pathway (Fig. S2B).

**Figure 2:**
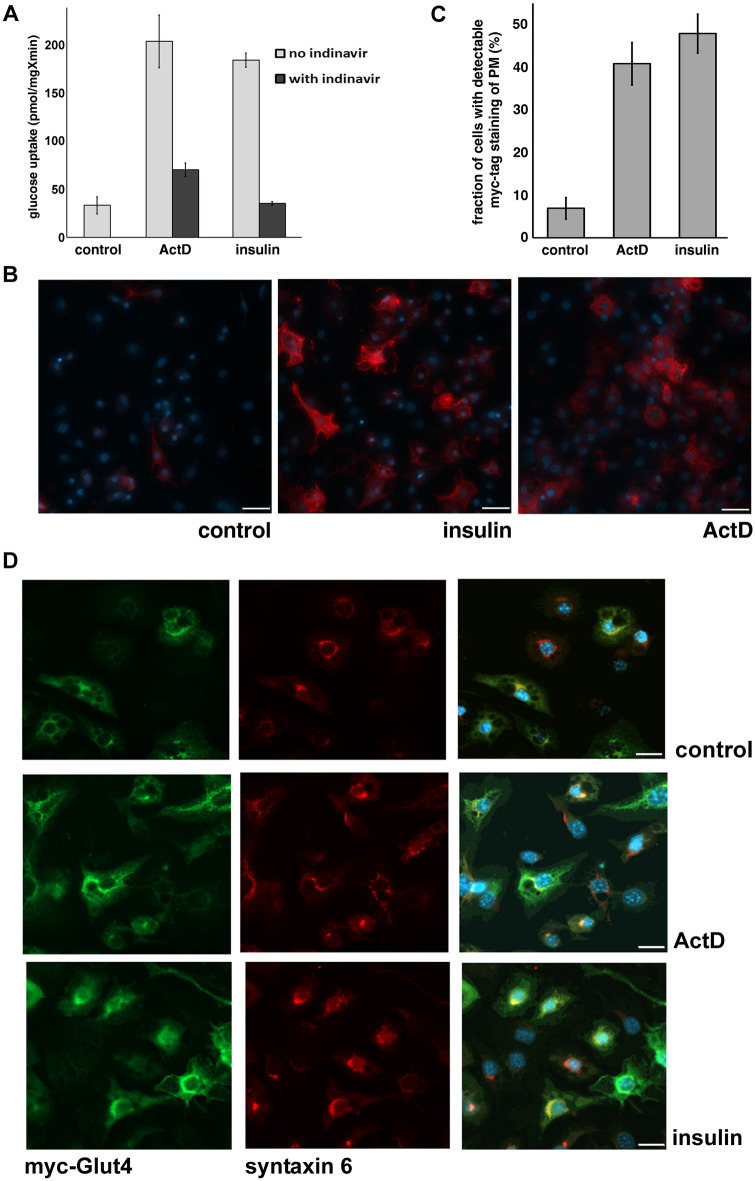
Actinomycin D-dependent upregulation of glucose uptake in adipocytes is mediated by Glut4. Panel (**A**): Differentiated 3T3-L1 adipocytes were treated or not treated with ActD (5 µM) for 18 h or insulin (100 nM) for 15 min and assayed for glucose uptake. Indinavir (50 µM) was added to the indicated samples 4 h before the glucose uptake assay and preserved throughout the assay. The error bars represent standard deviations. Panel (**B**): Differentiated 3T3-L1 adipocytes stably expressing myc_7_-Glut4 were treated or not treated with ActD (5 µM) for 18 h or insulin (100 nM) for 15 min, fixed and stained with anti-myc monoclonal antibody, Alexa Fluor 594-conjugated goat anti-mouse secondary antibody and DAPI without permeabilization. Representative images were obtained at the same exposure. The space bar corresponds to 50 µM. Panel (**C**): Quantification of results shown in panel B. The error bars represent standard deviations. Panel (**D**): Adipocytes stably expressing myc_7_-Glut4 were treated or not treated with ActD (5 µM) for 18 h or insulin (100 nM) for 15 min, fixed, and permeabilized. myc_7_-Glut4 was visualized with rabbit anti-myc antibody and Alexa Fluor 488-conjugated goat anti-rabbit antibody. Syntaxin 6 was stained with monoclonal anti-syntaxin 6 antibody and Alexa Fluor 594-conjugated goat anti-mouse antibody**.** Representative images were obtained at the same exposure. The space bar corresponds to 20 µM.

To determine whether ActD-induced increase in glucose uptake is mediated by Glut4, we have incubated adipocytes with a specific inhibitor of Glut4, indinavir^17^, as is shown in Fig. 2A, indinavir suppresses ActD- and insulin-stimulated glucose uptake with the same potency suggesting that both processes are mediated by Glut4. To confirm this result, we have used 3T3-L1 adipocytes stably expressing Glut4 tagged with seven myc epitopes in the first extracellular loop^15^. In non-permeabilized cells, only myc_7_-Glut4 that is integrated into the plasma membrane is accessible to anti-myc antibody. Figures 2B,C shows that both ActD and insulin induce translocation of myc_7_-Glut4 to the cell surface. Of note, that ActD does not affect the general structure of a major intracellular Glut4-containing compartment, the TGN^18–20^, marked with syntaxin 6 (Fig. 2D). Therefore, accumulation of Glut4 at the plasma membrane is unlikely to be explained simply by disintegration of the TGN upon ActD treatment.

**Figure 3:**
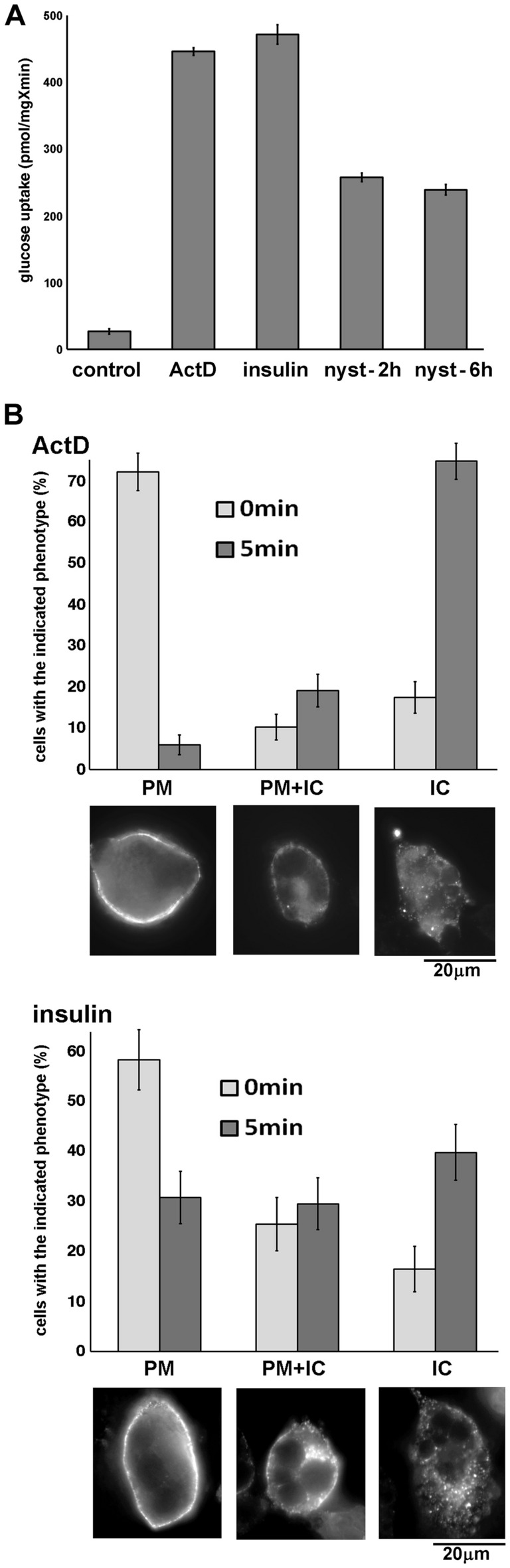
Efficient endocytosis of Glut4 in Actinomycin D-treated adipocytes. Panel (**A**): Glucose uptake in differentiated 3T3-L1 adipocytes treated or not treated with ActD (5 µM) for 18 h, insulin (100 nM) for 15 min, or nystatin (50 µg/ml) for 2 h and 6 h. The error bars represent standard deviations. Panel (**B**): Endocytosis of myc_7_-Glut4 in adipocytes treated with ActD (5 µM) for 18 h and insulin (100 nM) for 15 min was measured as described in *Materials and Methods*. Briefly, anti-myc monoclonal antibody was added for 1 h on ice to adipocytes stably expressing myc_7_-Glut4. Then, cells were either fixed with 4% formaldehyde (0 min), or transferred to pre-warmed DMEM for 5 min at 37 °C before fixation (5 min). Fixed cells were stained with Alexa Fluor 594-conjugated anti-mouse secondary antibody. PM—the antibody is almost exclusively associated with plasma membrane; IC—intracellular localization of the antibody, no detectable association with the plasma membrane; PM + IC—mixed phenotype. The error bars represent standard deviations. Representative images of cells with attributed phenotypes are shown at the bottom.

Redistribution of Glut4 from its intracellular compartment(s) to the plasma membrane may be caused either by activation of Glut4 exocytosis or by inhibition of its endocytosis. Indeed, an inhibitor of Glut4 endocytosis, nystatin^21^, increases glucose uptake in adipocytes. However, the effect of nystatin on glucose uptake is significantly smaller, than the effect of either insulin or ActD (Fig. 3A) suggesting that both insulin and ActD increase exocytosis of the transporter.

To further explore a potential effect of ActD on Glut4 endocytosis, we have analyzed internalization of myc_7_-Glut4 in insulin- and ActD treated cells. After ActD or insulin treatments, adipocytes were incubated with monoclonal anti-myc antibody for 1 h on ice. Under these conditions, internalization of Glut4 is suppressed, and the antibody will bind only to extracellular myc epitopes of tagged Glut4 at the plasma membrane. As is seen in Fig. 3B, this is observed in 60–70% of either insulin- or ActD-treated adipocytes (0 min conditions). Other cells demonstrate at least partial intracellular localization of myc_7_-Glut4 which may reflect an incomplete inhibition of internalization, or disruption of the plasma membrane upon cooling of adipocytes, or some other technical limitations of the approach.

Increasing the temperature to 37 °C for 5 min (5 min conditions in Fig. 3B) results in rapid internalization of myc_7_-Glut4 from the plasma membrane, leaving only about 30% of the insulin-treated and 5% of ActD-treated cells with the exclusive plasma membrane antibody staining. Correspondingly, in 40% of insulin-treated and in 70% of ActD-treated adipocytes the antibody has been completely internalized (Fig. 3B). These results suggest that endocytosis of Glut4 proceeds more efficiently in ActD-treated cells in comparison to insulin-treated cells and are consistent with earlier publications showing an inhibitory effect of insulin on Glut4 endocytosis in adipocytes (reviewed in^22^).

**Figure 4:**
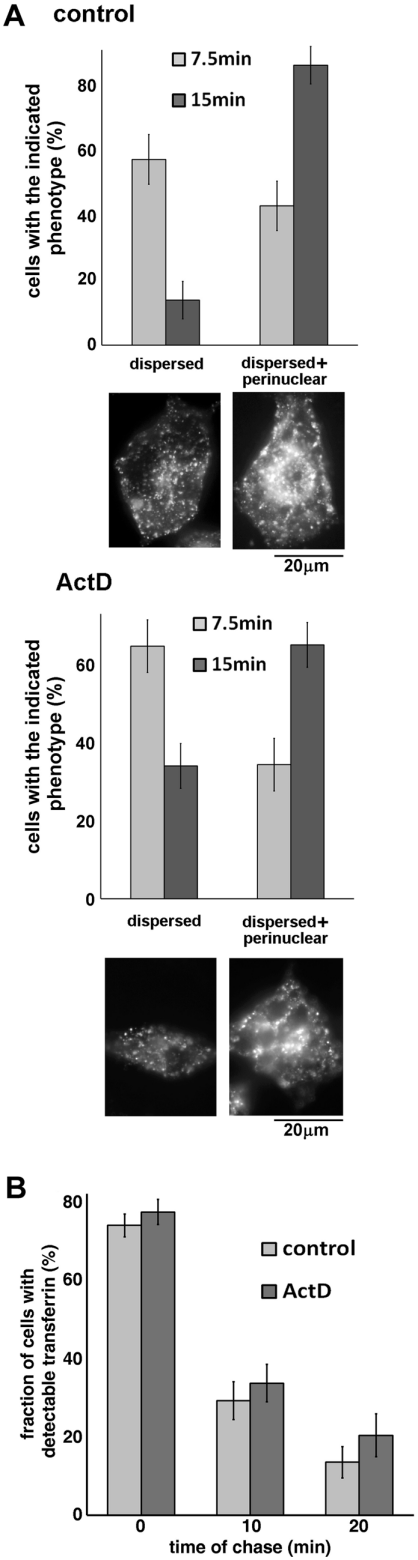
Actinomycin D does not affect recycling of transferrin in 3T3-L1 adipocytes. Panel (**A**): 3T3-L1 adipocytes were pre-treated or not with ActD (5 µM) for 18 h, and endocytosis of transferrin was measured for 7.5 and 15 min at 37 °C as described in *Materials and Methods*. Dispersed—no preferential localization of puncta in the perinuclear area; dispersed + perinuclear − significant fraction of puncta is localized in the perinuclear area. The error bars represent standard deviations. Representative images of cells with attributed phenotypes are shown at the bottom. Panel (**B**): Exocytosis of transferrin was measured in 3T3-L1 adipocytes treated or not treated with ActD (5 µM) for 18 h as described in “Materials and methods” section. The error bars represent standard deviations.

Endocytosis of Glut4 in basal adipocytes is difficult to study due to the very low abundance of the transporter at the plasma membrane in unstimulated cells. Therefore, we have tested the effect of ActD on endocytosis of transferrin, which shares the same clathrin- and AP-2-mediated endocytic pathway as Glut4^21–23^. Our analysis did not show any significant difference in endocytosis of Alexa Fluor 647-conjugated mouse transferrin between ActD-treated and non-treated adipocytes (Fig. 4A). In either case, after 7.5 min, Alexa Fluor 647-transferrin was found primarily (i.e. in ~ 60% of cells) in peripheral early endosomes. After 15 min, most Alexa Fluor 647-transferrin reaches perinuclear recycling endosomes. Of note, we did not observe any effect of ActD on the general intracellular distribution of Alexa Fluor 647-transferrin. This suggests that incubation with ActD does not lead to significant changes in the major intracellular membrane compartments traversed by recycling Glut4—endosomes (marked with Alexa Fluor 647-transferrin, Fig. 4A) and TGN (marked with syntaxin 6, Fig. 2D). Together, the data in Figs. 3 and 4A indicate that the effect of ActD on the plasma membrane accumulation of Glut4 can be attributed primarily to an increase in exocytosis of the transporter.

Importantly, ActD has no effect on exocytosis of transferrin from 3T3-L1 adipocytes. In this experiment (Fig. 4B), ActD-treated and untreated (control) adipocytes were loaded with Alexa Fluor 647-transferrin for 4 h, washed, and chased for 10 and 20 min at 37 °C. We did not detect any statistical difference in the number of Alexa Fluor 647-transferrin-positive cells between the two groups suggesting that the ActD-sensitive component is specifically involved in exocytosis of the IRVs.

**Figure 5:**
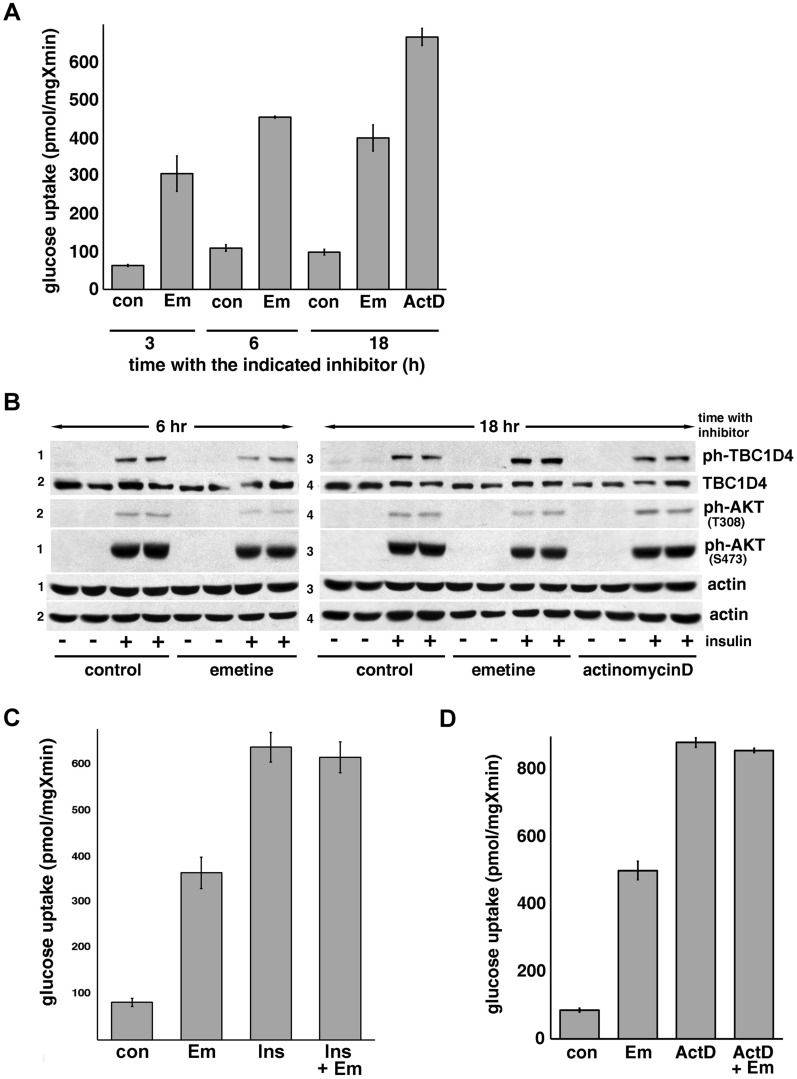
Emetine increases glucose uptake without engaging the insulin signaling pathway. Panel (**A**): differentiated 3T3-L1 adipocytes were incubated without (con) or with emetine (Em, 20 µM) and ActD (5 µM) for the indicated amount of time and assayed for glucose uptake. The error bars represent standard deviations. Panel (**B**): adipocytes were treated with emetine and ActD similar to samples in Panel (**A**), and the lysates from duplicate wells were analyzed by Western blotting. The panel represents a combined image of four different gels as indicated on the left. Loading controls (actin) shows equal loading and exposures. Panel (**C**): differentiated 3T3-L1 adipocytes were incubated without (con) or with emetine (Em, 20 µM, 6 h) and subjected to the glucose uptake assay in the absence and in the presence of insulin (100 nM, 15 min). The error bars represent standard deviations. Panel (**D**): Differentiated 3T3-L1 adipocytes were incubated in the absence (con) and in the presence of emetine (Em, 20 µM, 6 h), ActD (5 µM, 18 h), and combination of the drugs (5 µM ActD for 18 h and 20 µM of emetine for the last 6 h) as indicated and assayed for glucose uptake. The error bars represent standard deviations.

The effect of ActD on Glut4 translocation and glucose uptake may be associated with the depletion of a relatively short-lived protein(s) that, in basal adipocytes, acts as an intracellular anchor of the IRVs. To test this possibility, we examined the impact of the inhibitor of protein translation, emetine and found that it also increases glucose uptake in 3T3-L1 adipocytes (Fig. 5A) without affecting intracellular levels of Glut4^24^. Interestingly, emetine is less effective in activating glucose uptake in adipocytes, than ActD despite being more efficient in inhibition of global protein synthesis (Fig. S3). This may be explained by an adverse action of emetine on functionally important proteins, other than Akt and TBC1D4 as neither emetine nor ActD change total levels or phosphorylation of TBC1D4 in adipocytes (Fig. 5B). Similarly to ActD, emetine does not interfere with the effect of insulin on phosphorylation of Akt or TBC1D4 (Fig. 5B; for original files see Fig. S4), and the effects of emetine and insulin on glucose uptake are not additive (Fig. 5C). In addition, emetine does not potentiate the effect of ActD on glucose uptake (Fig. 5D). Notably, the effect of emetine on glucose uptake reaches its maximum in 3–6 h, i.e. emetine works faster than ActD. This difference may be attributed to the half-life of relevant mRNA(s) and protein(s). In general, data shown in Fig. 5 suggest that ActD and emetine act via the same mechanism, and that this may involve depletion of the same protein component(s).

**Figure 6:**
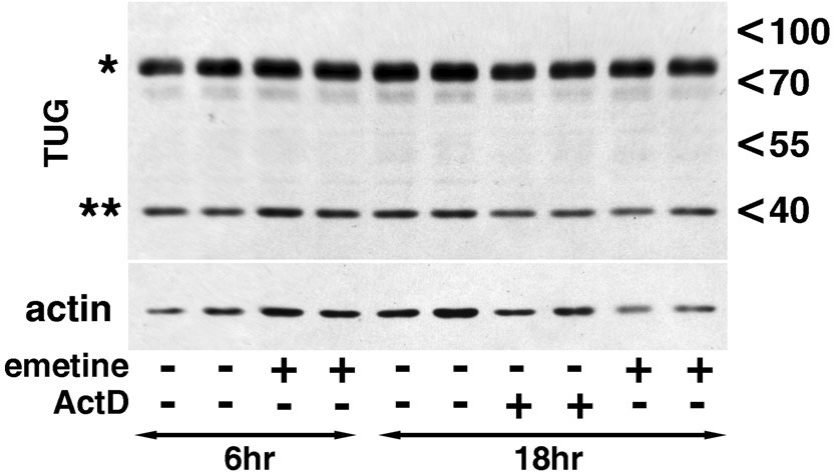
Actinomycin D and emetine do not affect proteolysis of TUG. Differentiated 3T3-L1 adipocytes were incubated in the absence and in the presence of emetine (20 µM) or ActD (5 µM) as indicated and the lysates from duplicate wells were analyzed by Western blotting. The asterisks mark the position of the full-length TUG (*) and its C-terminal proteolytic fragment (**). The positions of molecular weight markers are shown on the right.

One well-characterized protein that functions as an intracellular anchor for the IRVs is TUG. Since insulin triggers proteolysis of TUG by the protease USP25m via a PI 3 kinase-independent TC10α-PIST pathway^11–13^, this protein represents a good candidate for the putative ActD- and emetine-sensitive inhibitor of the IRV traffic revealed in our study. However, we have not detected any depletion of total (Fig. 6; for original files see Fig. S5) or membrane-associated (not shown) TUG under our experimental conditions.

## Discussion

The best studied routes of insulin signaling to the IRVs involve Akt-TBC1D4^8,9^ and TC10α-PIST-TUG^10,13^. We have shown that inhibition of protein biosynthesis stimulate translocation of Glut4 and glucose uptake in adipocytes in an apparent Akt- and TUG-independent fashion. Our results do not negate the role of the established signaling mechanisms, but rather uncover a novel aspect of the “Glut4 pathway”. Specifically, the data demonstrate that the intracellular retention of Glut4 requires de novo RNA and protein biosynthesis.

Currently, there are two general models that describe insulin-stimulated translocation of Glut4 (reviewed in^25^). According to one, “dynamic”, model, Glut4 slowly cycles between its intracellular compartments and the plasma membrane with insulin somehow increasing its exocytic rate and, possibly, slowing down its endocytosis. An alternative, “static”, model suggests that, under basic conditions, fusion of the IRVs with the plasma membrane is arrested by an as yet unknown mechanism and insulin lifts this block possibly by removing or inactivating a putative “intracellular anchor” of the IRVs.

One way to interpret our data is to suggest that there is an additional yet unidentified protein that is involved in intracellular anchoring of the IRVs. Under basal conditions, this protein should have relatively short half-life (less than 3 h as is indicated by data shown in Fig. 5A). In other words, it should undergo rapid degradation and be continuously replenished by de novo biosynthesis. Since ActD and emetine do not increase insulin-stimulated glucose uptake, we suggest that the insulin signaling pathway involves this ActD/emetine-sensitive anchor. It should be located downstream from Akt/TBC1D4 and TUG, as neither phosphorylation of Akt/TBC1D4 nor proteolytic cleavage of TUG is affected by the inhibitors. Insulin may inactivate this anchor either by phosphorylation via the PI 3 kinase/Akt-dependent mechanism or by controlled proteolysis via a PI 3 kinase-independent TC10α-PIST pathway. It is also possible that insulin acts to stimulate degradation of this putative anchor via Akt-dependent proteolysis^26^. At present, it is difficult to distinguish between these models as standard experimental methods, such as protein ablation and over-expression, represent major intrusions into adipose proteostasis that can tip the balance between phosphorylation and proteolysis or even totally mask the regulatory input of either pathway thus generating controversies.

In basal adipocytes, virtually all “movable” Glut4 is pre-sorted into the slow-recycling IRVs^27^. Upon insulin stimulation, these IRVs are mobilized directly to the plasma membrane, as assessed using total internal reflection fluorescence microscopy^28^. Thus, no Glut4 sorting is required for the initial translocation event. It is feasible that after the first round of translocation, sorting into the slow pathway (for example, formation of the IRVs) may be compromised. In this scenario, ActD and emetine should have at least two independent targets: initial translocation followed by impaired sorting, which is less likely from our standpoint. Thus, we think that our results are more consistent with the “static” model of IRV retention.

If there is an additional, short-lived protein that is required for intracellular anchoring of the IRVs, then how might such a protein be identified? siRNA- or CRISPR/Cas9-based screening combined with proteomic approaches could be the most direct and unbiased way to do this but would likely be technically challenging. Alternatively, a candidate approach can be taken. The effect of ActD and emetine is similar to that of TUG knockdown or deletion^12,29,30^. In both instances, little or no further effect of insulin was observed. The specificity of the effect for GLUT4, and not for endosomal proteins such as transferrin receptor, is also similar. Therefore, it is likely that ActD and emetine regulate the same vesicles that are trapped intracellularly by intact TUG, and that are mobilized upon insulin-stimulated TUG cleavage. In deletion analyses using transfected cells, the TUG C-terminus was required for Glut4 intracellular retention^11^. Therefore, an alternative approach would be to test candidates and/or to screen for proteins that bind the TUG C-terminus.

Can the effect of ActD and emetine on glucose uptake be attributed solely to increased translocation of Glut4? At present, this question is difficult to answer since, for technical reasons, the correlation between glucose uptake and Glut4 translocation has been established qualitatively rather than quantitatively^31^. Within the frame of this ambiguity, various possibilities can be discussed. For example, it has been proposed that during steady state conditions of maximal insulin stimulation in muscle, some control over the overall rate of glucose uptake is shifted to the 6-phosphorylation of glucose by hexokinase, which traps glucose intracellularly^32^. Thus, one can envision that inhibitors might somehow enhance the phosphorylation of 2-deoxyglucose by hexokinase. However, we measure 2-deoxyglucose uptake using an incubation period of only 5 min. Under these conditions, any significant input of hexokinase is unlikely. Furthermore, incubation with ActD or emetine should, if anything, decrease intracellular levels of hexokinase; such an effect would not increase glucose uptake.

In recent years, most research efforts directed towards understanding insulin resistance have focused on potential defects in proximal steps of the canonical insulin signaling pathway^33^. In particular, Ser/Thr phosphorylation of IRS1 counter regulatory to insulin signaling has been observed in multiple experimental contexts^34^. However, these upstream IRS phosphorylation events do not necessarily correlate with decreased glucose uptake^35,36^. Moreover, other studies show that type 2 diabetic patients and their first-degree relatives can have impaired glucose transport despite normal Akt phosphorylation (see, for example^37–39^). In addition, genetic ablation of Akt is not sufficient to induce insulin resistance or prevent glucose uptake in skeletal muscle^40^. We believe therefore, that identification of the downstream trafficking inhibitor proposed in our study may help to explain the molecular nature of insulin resistance and promote development of novel treatment modalities.

## Supplementary Information

Supplementary Figures.

## Data Availability

All data generated or analyzed during this study are included in this published article and its supplementary information files.
