# Supplementary material for: Inhibitors of RNA and protein synthesis cause Glut4 translocation and increase glucose uptake in adipocytes
Source: Sci Rep. 2022 Sep 19;12:15640. doi: 10.1038/s41598-022-19534-5 (PMC9485115; doi:10.1038/s41598-022-19534-5)
Supplement: Supplementary file 1 — Supplementary Figures. [file 41598_2022_19534_MOESM1_ESM.pdf]

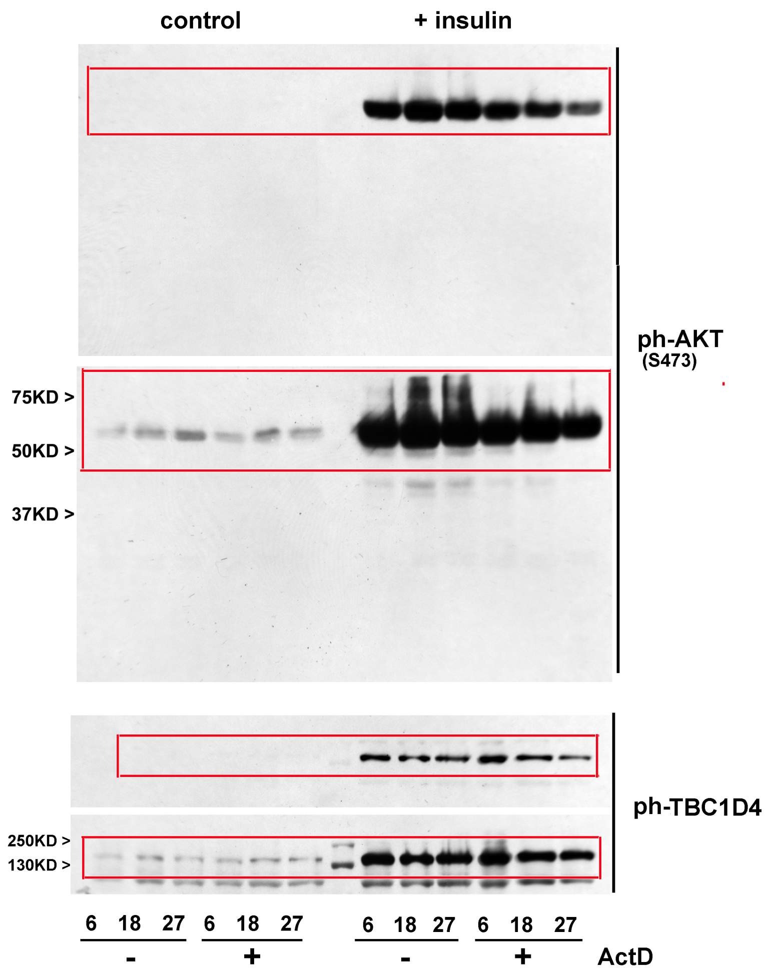

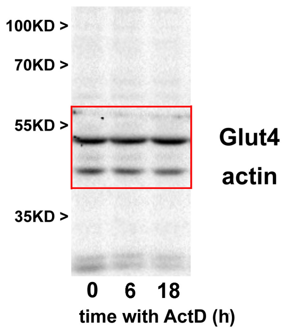

Figure S1. Unprocessed images shown in Fig. 1B and 1C. Red boxes denote cropped images.

Figure S1

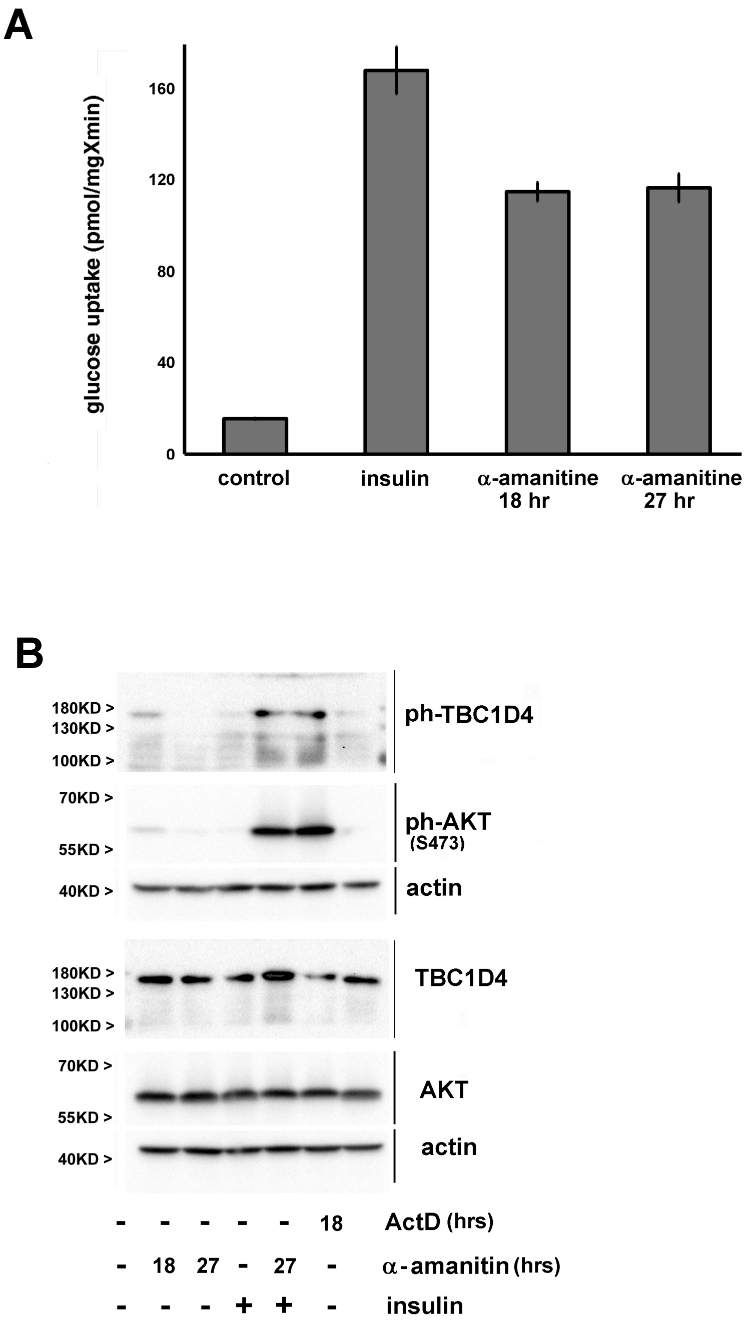

Figure S2. α-amanitine increases glucose uptake in adipocytes without engaging the insulin signaling pathway. α-amanitine (5 µg/ml) or insulin (100 nM) were added to differentiated 3T3-L1 adipocytes, and cells were assayed for glucose uptake (Panel A) or analyzed by Western blotting (Panel B). In panel B, ActD (5 µM) was added to last wells as a reference. The blot was cut, and the resulting 6 strips were hybridized with indicated antibodies; the images of the strips were uncropped. The error bars represent standard deviations.

Figure S2

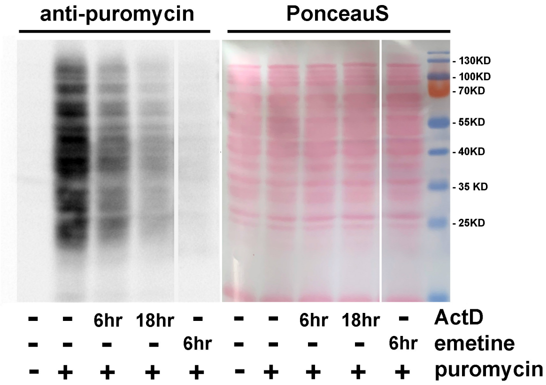

Figure S3. ActD and emetine suppress protein synthesis in 3T3-L1 adipocytes as measured by incorporation of puromycin. 3T3-L1 adipocytes were treated with ActD (5 μM) or emetine (Em, 20 μM) for the indicated amount of time. After that, puromycin (2 μg/ml) was added where indicated for 30 min, and cell lysates were analyzed by Western blotting with anti-puromycin antibody (left) and Ponceau S staining (right).

Figure S3

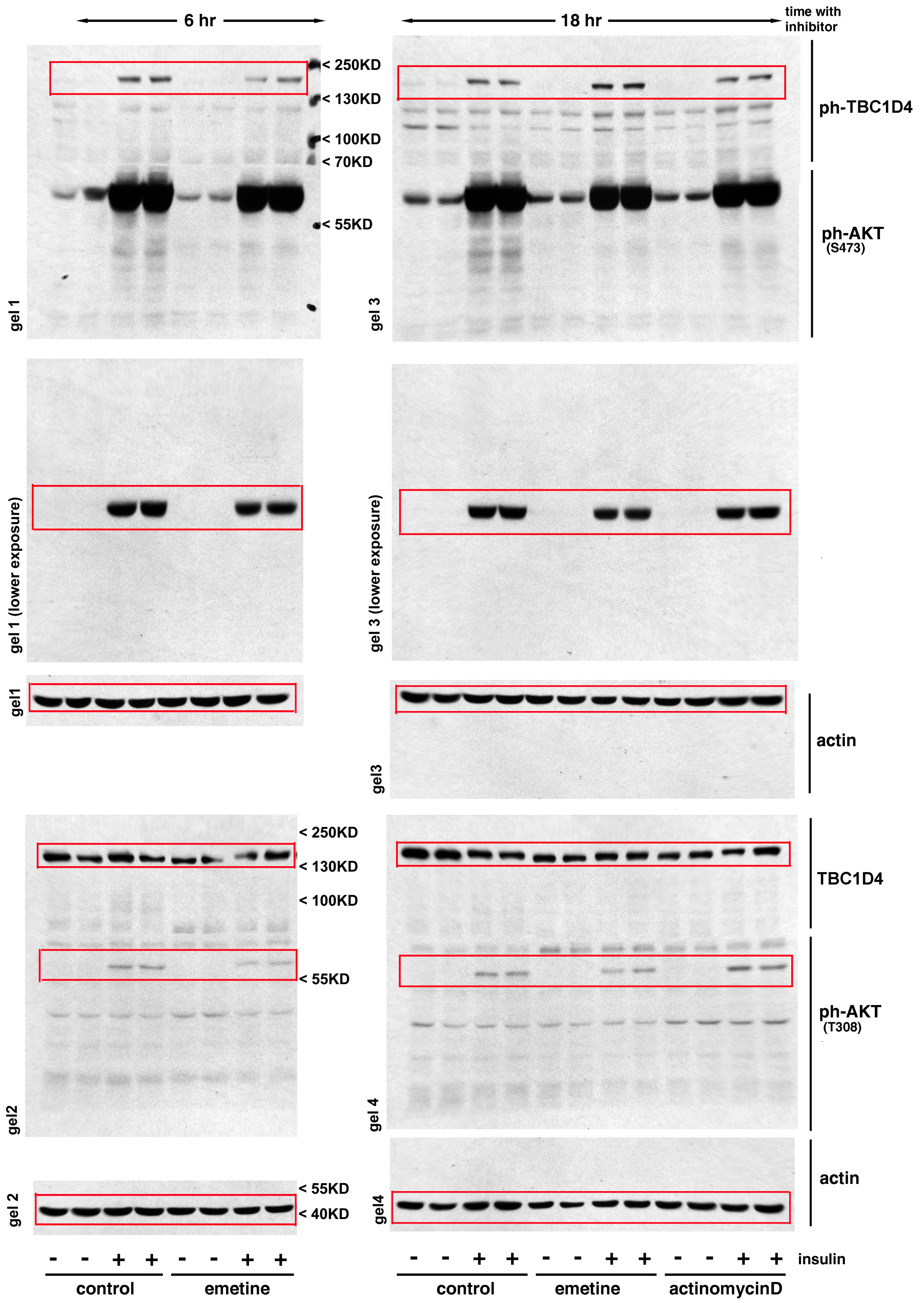

Figure S4. Unprocessed images shown in Fig. 5B. Red boxes denote cropped images.
Two exposures of ECL signals from ph-TBC1D4 and ph-AKT (S473) are shown.

Figure S4

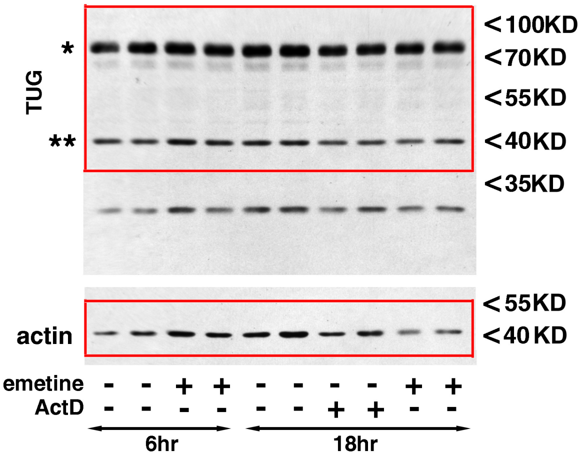

Figure S5. Unprocessed images shown in Fig. 6. Red boxes denote cropped images.

Figure S5
